# Elderly suicide trends in the context of transforming China, 1987–2014

**DOI:** 10.1038/srep37724

**Published:** 2016-11-25

**Authors:** Bao-Liang Zhong, Helen F. K. Chiu, Yeates Conwell

**Affiliations:** 1 Department of Psychiatry, The Chinese University of Hong Kong, Hong Kong SAR, China; 2 Affiliated Mental Health Center, Tongji Medical College of Huazhong University of Science and Technology, Wuhan, Hubei, China; 3 Department of Psychiatry, University of Rochester Medical Center, Rochester, New York, USA

## Abstract

In the context of rapid ageing, understanding the time-trend of elderly suicide (ES) could inform China’s efforts on suicide prevention. We examined time-trends in Chinese ES rates (ESRs) from 1987 to 2014, a period of profound social changes. Suicide rates by residence (rural/urban), gender, and 5-year age-group (65+) in 1987–2014 were provided by the Chinese Ministry of Health. Time-trends were analyzed with joinpoint analysis. The time-trend of national ESRs was downward (average annual percent change [AAPC] = −3.7, P < 0.001): 76.6/100000 in 1987 and 30.2/100000 in 2014. However, the time-trend of corresponding percentages of ESs among the total suicides was monotonically increasing (AAPC = 3.4, P < 0.001): 16.9% in 1987 to 41.2% in 2014. The time-trends in ESRs of both rural and urban men and women were decreasing, but only the rural trends were significant (P < 0.001). Rural-urban and male-female differences in ESRs were decreasing over time (slope = −4.2 and −3.0, P ≤ 0.006), but the rural-urban and male-female ESR differences in 2014 remained large (16.3/100000 and 9.8/100000, P < 0.001). While national ESRs decreased significantly during the past three decades, the current ESR remains high in China. Further, the age-pattern of Chinese suicide is transitioning to elderly predominance. ES, particularly rural ES, should be a public health priority in China.

In China, the past three decades have witnessed a sharp drop in the official national suicide rates, from 17.6 per 100000 in 1987 to 7.46 in 2014^1^,^2^. The unique pattern of rural and female predominance of suicide in China also changed dramatically; for example, the rural-to-urban and female-to-male ratios significantly declined from 2.8 and 1.4 in 1987 to 1.7 and 0.8 in 2014, respectively^1^,^2^. However, time-trend studies found that the changing patterns of suicide in China varied considerably between residences (rural/urban), genders, age-groups and even geographic regions^3^,^4^,^5^,^6^,^7^; specifically, the drop in suicide rates was more dramatic among younger age-groups, particularly young rural women, but was less pronounced in older age-groups, and even, in 2009–11, it was predicted that Chinese elderly suicide rates (ESRs), especially rural ESRs, were likely to go up again^5^,^8^,^9^.

China has experienced a remarkable demographic transformation over the past decades, with the proportion of over-65 population increasing from 5.5% in 1987 to 10.1% in 2014^10^. Until now, China has the largest elderly population (137.6 million) in the world^11^, and, by 2050, the elderly population will grow to 336 million, representing almost a quarter of the total Chinese population^12^. Because people born after the founding of new China had reached age 65 years in 2014, the elderly population growth rate will accelerate in the upcoming years^12^.

Similar to most countries in the world, China’s suicide rate generally increases with age and rate increases substantially after age 65 years in both men and women residing in rural and urban communities^5^,^6^,^13^. In 2013, the WHO Mental Health Action Plan 2013–2020 set a global target to reduce the suicide rate of each WHO member state by 10% by the year 2020^14^. Because suicide rates in young age-groups have already been very low in recent years^5^,^6^,^13^, there may be limited room for a further decline in rates of young Chinese. Considering the very high ESRs relative to other age-groups and the unprecedented population aging in China, elderly suicide (ES) prevention should be given greater priority for reducing Chinese suicides.

In parallel with China’s rapid development of a market-oriented economy in the last 30 years, many sociocultural changes have occurred: rapid urbanization and economic development, massive rural-to-urban migration, improved standards of living and healthcare, the significant shift in value from Confucian collectivism to individualism, and diminished traditions of filial piety and respect for elders^11^,^15^,^16^,^17^,^18^. Because suicide is often linked to complex social, economic and cultural factors^19^,^20^, it is expected that the ES pattern should have changed over time. Detailed information on the changing epidemiology of ES in the context of China’s significant macro-social changes might serve to inform the development of current and long-term national suicide prevention strategies. However, only two previous studies^3^,^5^ described time-trends in suicide rates of Chinese general population with a little attention on suicide of different age-groups of older adults (OAs), not OAs of all age-groups as a whole. Moreover, the two studies analyzed time-trends separately for two non-consecutive periods (1991–2000 and 2002–2011) and did not provide any trend data on residence, gender and age patterns of ES. Therefore, the changing patterns of ES in China remain largely unknown.

This study aimed to examine the temporal trends in ESRs in China from 1987 to 2014, a period when China’s suicide mortality data were publicly available. Based on the declining trends in ESRs of several old age-groups reported in the two studies mentioned above^3^,^5^, we hypothesized that ESRs of Chinese OAs, regardless of residence, gender and old age-group, have decreased and features of ES, such as rural-urban and male-female rate differences have also changed since 1987.

## Methods

### Data source

There is no complete nation-wide mortality surveillance system in China and, for many years, China’s suicide mortality statistics have been derived from a sample-based death cause surveillance system, the China’s Ministry of Health Vital Registration (MOH-VR) system^21^. The MOH-VR was formally established in 1987 and its sampling regions and population covered by the system have expanded since then. In 1987, the system covered 99 million people in more than 100 cities and counties, approximately 10% of the total Chinese population^22^. By 2012, the system included a total of 138 rural counties and 181 urban districts from 22 provinces of China and the population covered by the system had increased to 17% of the total population^23^. However, the MOH-VR data before 2013 are generally not representative of China’s mortality conditions because the system purposively sampled counties and cities with relatively good reporting mechanisms, primarily distributed in eastern and central China^23^,^24^. To ensure the sample representativeness, in 2013 the MOH extensively modified MOH-VR and established the Integrated National Mortality Surveillance System (INMSS), which retained about one third MOH-VR’s original surveillance sites and included over 500 new representative sites^23^. By 2014, the INMSS had included 605 surveillance sites from all 31 provinces covering 323.8 million people, roughly 24% of the total population^23^. The MOH-VR data, while not representative, are useful for suggesting trends in mortality, given the number of deaths covered^25^.

Cause of death was coded according to ICD-9 in 1987–2001 and ICD-10 in 2002–2014^23^. The data of MOH-VR/INMSS are based on death certificates completed by staff of local hospitals and submitted to local Centers for Disease Control and Prevention, which are then monthly reported to the MOH^23^. Suicide rates by gender for quinquennial age-groups for both rural and urban residents (Supplementary Table S1) were provided in the Annual Reports of Health Statistics of China (1987–2001)^26^, the Chinese Health Statistics Yearbooks (2002–2012)^27^ and China Health and Family Planning Statistics Yearbooks (2013–2014)^28^.

### Statistical analysis

In this study, OAs were persons aged 65 years and older. All ESRs were expressed as the number of suicides per 100000. We investigated changes in the time-trends of (1) national ESRs, (2) residence-gender-specific ESRs, (3) residence-gender-age-specific ESRs, and (4) percentages of ES deaths among national, rural and urban suicide victims, (5) national and gender-specific rural-urban ESR differences (RU-ESRDs, rural ESR minus urban ESR), and (6) national and residence-specific male-female ESR differences (MF-ESRDs, male ESR minus female ESR). For 1–4, joinpoint regression was employed. This statistical modeling technique is widely used to explain the relationship between time and mortality and percent by means of a segmented linear regression constrained to be continuous everywhere, particularly in those time-points where the slope of the regression function changes^29^. The annual percent change (APC) and its 95% confidence interval (CI) were used to characterize trends in ESRs and percentages. For 5–6, as joinpoint analysis is not applicable for negative values in RU-ESRDs and MF-ESRDs, we used linear regression to test their overall time-trends.

We primarily focused on time-trends of actual annual ESRs (crude rates), percentages of ES deaths and residence/gender differences in crude ESRs, because standardized ESRs might be insufficient to reflect the true level of OAs’ potential needs for suicide prevention. Given that residence-, gender- and age-specific suicide rates were already available, annual MOH-VR surveillance population data stratified by residence, gender and age were necessary for calculating annual ES counts and ESRs, which were then used for the joinpoint analysis. Unfortunately, for some unknown reasons, such data for 2001–2012 were not published by the MOH. More importantly, these population data can not be used directly even if they were available, as the MOH-VR population was a biased sample of the Chinese general population^24^. We therefore operationally defined 28 representative surveillance samples for 1987–2014, by constraining their sizes to be 10% of the total Chinese population of the corresponding years. Surveillance population data by residence, gender and age were calculated by directly applying the sex-by-age group by residence proportion data, provided in China’s Population Statistics Yearbooks^30^,^31^, to these surveillance samples of corresponding years. Consistent with previous studies^5^,^7^,^21^, persons living in towns and villages in the population statistics yearbooks are defined as rural population, while persons living in cities as urban population.

We first calculated the number of ESs of a specific year, by multiplying residence-sex-age-specific suicide rates by the number of OAs in the corresponding residence-sex-age-specific groups of the corresponding surveillance sample, and added them up to get the total count of ESs. The crude ESR for each corresponding year was generated through dividing total count of ESs by the total elderly number of the surveillance sample. ESRs for OAs aged 65–74 (young-old), 75–84 (old-old) and 85+ (oldest-old) years were calculated in the same manner. To exclude the impact of demographic changes on the temporal trends of ESRs, we also standardized the crude ESRs for the period of 1987–2014 to the residence-age-gender-specific population statistics from the 2010 National Census population^31^. Segmented regression and linear regression were performed using the Joinpoint Regression Program (version 4.3, US National Cancer Institute)^32^ and SPSS Statistics (version 20, IBM Corporation), respectively. The P-value for statistical significance is defined as a two-sided P < 0.05.

### Ethics

Secondary data analysis based on open statistics data do not require approval from the Research Ethics Committee System.

## Results

### Trends of the national ESRs, 1987–2014

**Figure 1:**
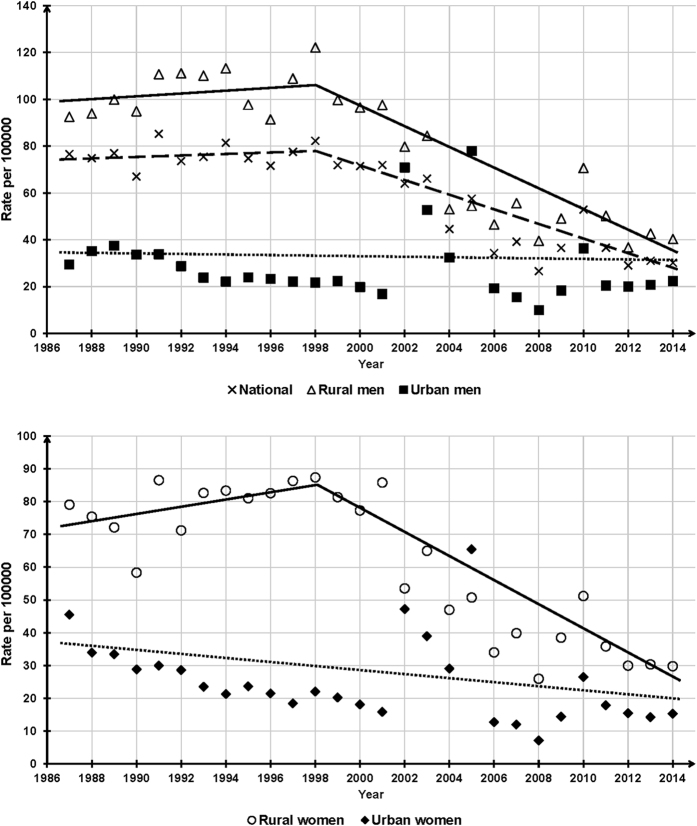
National and residence-gender-specific elderly suicide rates, China, 1987–2014.

The national ESR significantly decreased from 76.6 in 1987 to 30.2 per 100000 in 2014 and this downward trend remained significant if crude ESRs were standardized to the 2010 census population, with an Average APC (AAPC) of −3.7 (P < 0.001). Joinpoint analysis split the ESR trend into two segments, a gradual but insignificant increase from 1987 to 1998 (APC = 0.5, P = 0.60), and a significant reduction from 1998 to 2014 (APC = −6.0, P < 0.001) (Fig. 1 and Table 1).

### Trends of residence-gender-specific ESRs, 1987–2014

The changing patterns of ESRs of rural men and women were the same as that of the national ESR. ESRs of both genders slightly increased between 1987 and 1998, with an APC of 0.8 for men (P = 0.60) and 1.6 for women (P = 0.30), respectively, and dramatically decreased from 1998 onward, with a significant APC of −6.4 for men (P < 0.001) and −6.9 for women (P < 0.001), respectively. Overall, between the years 1987 to 2014, the growth rates in ESRs of rural men and women were −3.8% (95%CI: −4.8, −2.8) and −3.7% (95%CI: −4.9, −2.6) per annum, respectively (Fig. 1 and Table 1).

Urban men and women showed a slight declining trend in ESRs during the observation period, with an AAPC of −0.7 for men (P = 0.70) and −1.9 for women (P = 0.10), respectively. We did not identify any change points in the two urban trends (Fig. 1 and Table 1).

### Trends of residence-gender-age-specific ESRs, 1987–2014

**Figure 2:**
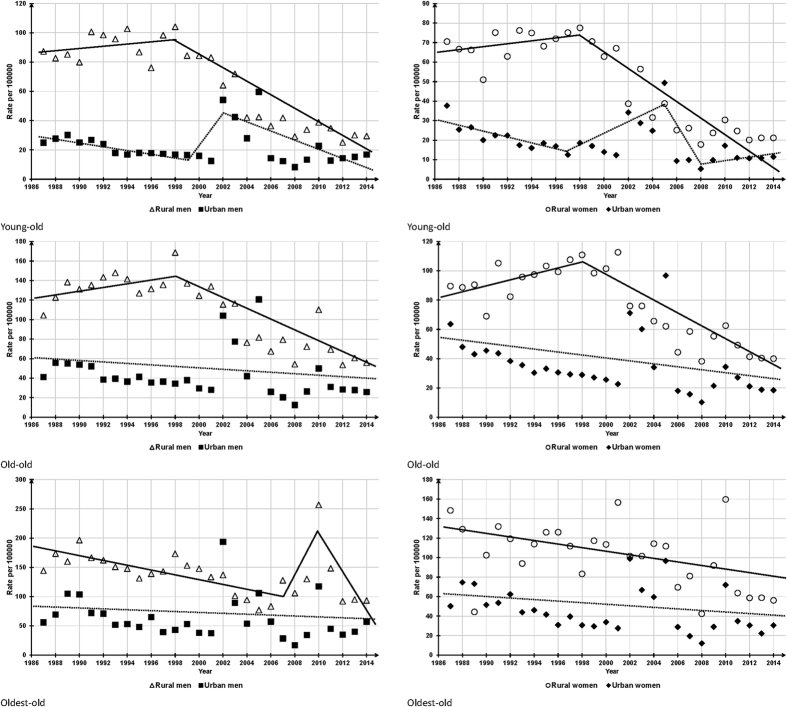
Residence-gender-specific elderly suicide rates, China, 1987–2014.

In rural China, young-old and old-old men and women all had similar changing patterns: between the years 1987 to 1998 and 1998 to 2014, ESRs slightly rose 0.8–2.3% per year (P = 0.10~0.50) and significantly decreased 5.8–8.8% per year (P < 0.001), respectively, leading to significant overall decreasing trends in ESRs (APC = −4.8~−3.3, P < 0.001). In the rural oldest-old group, females presented a significant and monotonically downward trend in ESRs (AAPC = −1.9, P = 0.005) and males presented a weak declining trend in ESRs (AAPC = −1.0, P = 0.23) with two significant segments: a steady decrease in 1987–2007 (APC = −3.2, P < 0.001) and a rapid decrease in 2010–2014 (APC = −22.5, P < 0.001) (Fig. 2 and Table 2).

In urban China, young-old, old-old and oldest-old males and females all presented a non-significant decreasing trend in ESRs (AAPC = −0.8~−2.2, P = 0.24~0.62). ESRs of urban young-old men had a significant reduction from 2002 to 2014 (APC = −10.7, P < 0.001), but there was a sudden rise from 1999 to 2002 (APC = 50.5%, P = 0.81). ESRs in urban young-old women significantly decreased 8.4% (P < 0.001) per year from 1987 to 1997, but significantly increased 14.1% (P < 0.001) per year during 1997–2005 (Fig. 2 and Table 2).

### Trends of percentages of ES deaths among national, rural and urban suicide deaths, 1987–2014

**Figure 3:**
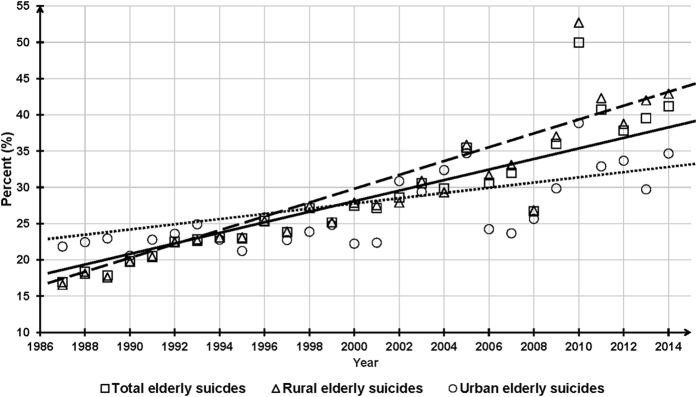
Percentages of elderly suicides among national and residence-specific suicide deaths, China, 1987–2014.

The national, rural and urban percentages of ESs among the total, rural and urban suicides were monotonically and significantly increasing over time, with an AAPC of 3.4, 3.6 and 2.0 (all P < 0.001), respectively. The national percentages of ESs increased from 16.9% in 1987 to 41.2% in 2014 (Fig. 3).

### Trends of national and gender-specific RU-ESRDs, 1987–2014

**Figure 4:**
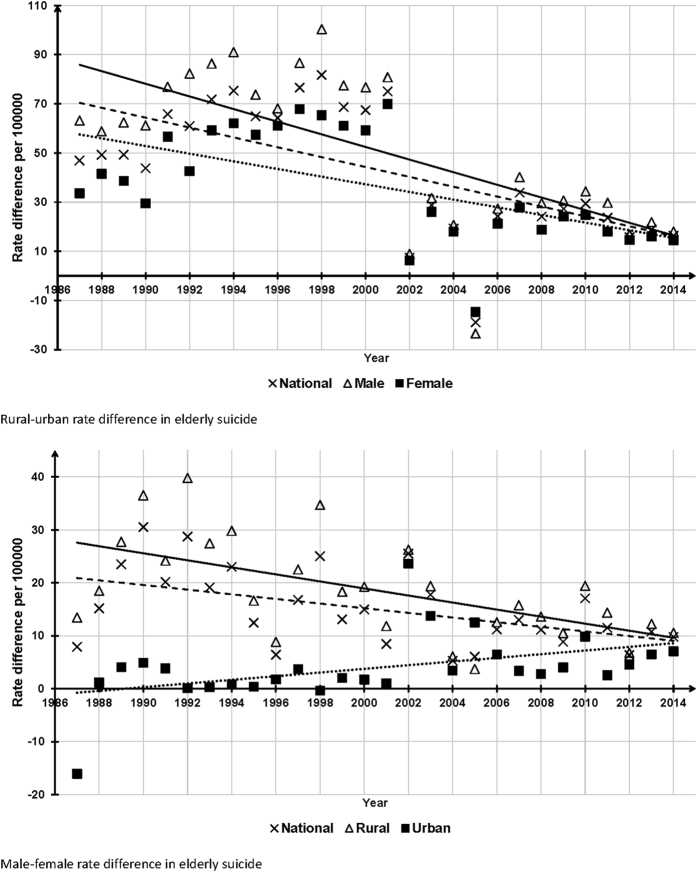
National and gender-specific rural-urban and national and residence-specific male-female differences in elderly suicide rates, China, 1987–2014.

The national, male and female rural-urban differences in ESRs all showed significantly decreasing trends over the whole observation period. Their corresponding estimated slope coefficients (βs) from linear regression models were −4.2, −4.8, and −3.6 (all P < 0.001), respectively (Fig. 4). However, the 2014 national RU-ESRD was still significantly higher than 0 (16.3 per 100000, 95%CI: 14.3, 18.3, P < 0.001).

### Trends of national and residence-specific MF-ESRDs, 1987–2014

The overall time-trends of national and rural male-female differences in ESRs were downward and significant (β = −3.0, P = 0.006 and β = −3.7, P = 0.001), but the overall time-trend of urban MF-ESRDs was upward and significant (β = 2.5, P = 0.018) (Fig. 4). The 2014 national MF-ESRD remained significantly higher than 0 (9.8 per 1000000, 95%CI: 7.9, 11.6, P < 0.001).

## Discussion

Due to the markedly high suicide rates in Chinese rural young women in the 1990 s, much research and public attention was paid to suicide of young adults^33^,^34^,^35^ while ES has been neglected in China. To the best of our knowledge, this is the first study that reports long-term trends of ESRs based on China’s largest regular national mortality monitoring system during a long period spanning from 1987 to 2014. Although the MOH-VR surveillance sample is not representative of the Chinese population, the residence-gender-age-specific suicide rates are generally considered as unbiased point estimates of the true prevalence of suicide in China^5^,^7^,^21^,^36^. In the absence of representative surveillance population data, a representative subsample of the Chinese general population of a specific year was used. Given the very large sample (over 100 million), this approach provided a fairly accurate and reliable estimates of the prevalence of ESRs, thereby making our findings more generalizable.

The main findings of this study were: (a) there was a significant downward trend in the national ESR, with the year 1998 as the turning point towards rapid decline; (b) ESRs of rural men and women had the same changing patterns and turning points as those of the national population, however, ESRs of urban men and women did not present significant declining trends; (c) the changing patterns of rural young-old and old-old men and women and urban old-old and oldest-old men and women were the same as those of rural men and women and urban men and women as a whole, respectively; (d) a significantly and monotonically upward trend was observed in national percentages of ESs among the total suicide deaths; and (e) a significant downward trend was presented in national RU-ESRDs and MF-ESRDs, however, such rural-urban and male-female differences remained statistically significant in 2014.

As expected, ESRs in China have experienced a very sharp decrease in the past 28 years, but the downward trends in urban OAs were not significant, indicating that significant drops in rural ESRs are the main driving force of the decreased national ESRs. Nevertheless, the directionality and pace of changes were not constant over time, as shown in Fig. 1, national and rural ESRs reversed from slow increase to rapid decease at 1998. In the further analyses by age-groups, though rural oldest-old and urban young-old adults exhibited different patterns of changes in ESRs in contrast to their corresponding residence-gender-groups, the overall time-trends of all age-groups were similar to the residence-gender-group they belonged to.

The overall downward trend in ESRs in this study is consistent with two previous reports on suicide trend of China^3^,^5^. Nevertheless, patterns of changes in ESRs found in our study are quite different according to residence-gender-age groups and particular periods. For example, in 1991–2002, Chinese ESRs significantly decreased in urban OAs, but not in rural OAs^3^, and between 2002 and 2011, Chinese ESRs significantly decreased in young-old urban adults and rural men, and rural women aged 65–79 years, but not in 75+ urban adults and rural men, and 80+ rural women^5^. Unlike these findings, we found only rural ESRs dramatically decreased between 1998 and 2014 and the overall downward trends were significant in nearly all gender-age-groups of rural OAs, but not urban ESRs, either in whole or in sub-groups. Differences in observation periods, trend analysis methods, and age grouping criteria between studies might explain these various changing patterns.

It is remarkable that a drop of as much as 60.6% in the national ESR was observed from 1987 to 2014, because China had mounted few suicide specific prevention programs during that interval. Considering the involvement of socioeconomic environmental risk factors in the etiology of suicide, the profound socioeconomic changes in recent decades are more likely to have contributed to the decrease in China’s ESR. Researchers have ascribed the reduction in China’s national suicide rate to rapid economic growth, improved living standards, rural-to-urban migration of rural young labors, economic independence of rural young women, and more accessible health services^3^,^4^,^5^,^7^,^20^. It has been reported that chronic illness, family discord, financial difficulty and mental disorder are the major factors associated with ES in China^37^. In our study, the drop of the national ESR was mainly caused by declines in rural ESRs, so we speculate that several factors may contribute to the decreasing ES. These include: improved financial condition resulting from rural young labors’ migration to work in cities (who also send remittances to their older parents), more accessible health services (including mental health services) and better social welfare, as well as pesticide control measures. For example, China established the New Rural Cooperative Medical Scheme to reduce the financial burden of healthcare on rural residents in 2003^38^, abolished the agricultural tax in 2006^39^, motivated and trained village doctors to provide a package of basic public health services in 2009 (including chronic disease management and elderly health)^40^, and established the New-type Rural Endowment Insurance system in 2009 to enhance rural old-age social and economic security^41^. Furthermore, because ingestion of pesticides is the most common suicide method for Chinese OAs^37^, reduced access to pesticides may be another explanation for the decreased ESRs. In 1997, China established national regulations to supervise and manage the production, sale and use of pesticides. From 1999 to 2007, this regulation was revised to strengthen supervision of their use by consumers as well. By 2014, a total of 33 types of highly lethal pesticides were forbidden to be produced or sold by the Ministry of Agriculture^42^. It is likely that China’s control on pesticide use since 1997 is one important contributing factor for the reduced ESR, as the upward trend in Chinese ESRs was immediately reversed at 1998 in our analysis. In sum, although all the above-mentioned measures since 1997 are not specific for suicide prevention, they may have contributed to the declining ESR, in particular rural ESR.

Compared to the cut-off rates that were used to rank countries by ESRs, provided by a cross-national comparison study^43^, China’s 2014 ESRs of males and females fall into the second highest group and the highest group, respectively. Therefore, despite significant decreases in the national ESRs, China’s ESR is still relatively high in 2014 in the world. Further, percentages of ESs among the total Chinese suicide deaths had a 2.4-fold increase from 1987 to 2014, while corresponding percentages of OAs among the total Chinese population only had a 1.8-fold increase^10^. This phenomenon should be the result of a much more slowly decreasing rate in ESRs than suicide rates of younger age-groups and the rapid aging of the Chinese population, indicating that ESRs are disproportionately high and the relative importance of ES is progressively increasing in China.

In addition, although we found significant decreasing trends in national RU-ESRDs and MF-ESRDs, the 2014 national RU-ESRD and MF-ESRD were still significantly higher than 0, indicating the rural-urban and male-female gaps in ESRs still exist. The significant RU-ESRD may be attributed to China’s long-lasting Rural-Urban Dual Society System, which was established in 1950 s by the government and used to determine the allocation of social resources based on citizens’ rural/urban household registration^18^. Compared with residents registered as urban, those registered as rural have less access to job opportunities, housing subsidies, free education, medical services, and old-age pensions^17^. Owing to the uneven economic development between rural and urban areas, coupled with this discriminatory dual system, inequalities in health care and social welfare between rural and urban may result in the rural-urban differentials in Chinese ESRs.

China was the only country in which the overall female suicide rate exceeded male rate in the 1990 s and early 2000 s, but this gender pattern was only true for rural young adults^44^. The higher ESRs in males versus females in our study are consistent with most other countries^14^. Significant opposite time-trends in rural and urban MF-ESRDs were demonstrated in this study, thus the significant downward trend in national MF-ESRD was also caused by the decreasing rural MF-ESRDs. The improving social status of Chinese rural women is a possible explanation for the decreasing rural MF-ESRD, but it is unclear why urban MF-ESRDs increased over time; perhaps the increasing urbanization of China is associated with more urban male ESs^44^.

Limitations of this study should be noted. First, information on trends of ESRs by suicide methods could greatly inform ES prevention policy making, but the data source did not contain these data. Second, under-reporting and misclassification are two common problems in the VR systems of developing countries such as China^5^, but these two potential issues have not been adjusted for in our analysis due to the lack of such data, potentially leading to an underestimation of ESRs in this study. However, considering that data quality of the MOH-VR have improved over time^23^, the problem of under-estimation may be more serious during earlier years. If we had data with which to make adjustments for under-estimation, our findings on the downward trends would be more pronounced. Third, a complete death registration system in China is still not feasible, owing to the large size of Chinese population. Therefore, we have to rely on rates computed from a sample of 10–24% of the total national population to examine the time-trends in ESRs. Strictly speaking, these residence-gender-age-specific suicide rates from the MOH-VR should have sampling errors, which are omitted in our analysis. However, because the surveillance samples are very large (as discussed above), it is methodologically acceptable to ignore errors in these crude rates^45^.

In summary, China’s suicide age-pattern is transitioning to elderly predominance due to the increasing percentages of ESs among total suicides. The residence- and gender-patterns of ES continue to reflect rural and male predominance, although rural-urban and male-female gaps in ESRs are narrowing over time. Significant downward trends in China’s national and rural ESRs have been observed since 1998, but the national ESR in 2014, particularly rural ESR, remains high relative to other countries worldwide. In addition to the overall unchanged urban ESRs, the continuously increasing percentages of ESs and rapid aging of the Chinese population, ES, particularly in rural areas, should be addressed as a major public health concern in China going forward.

## Additional Information

**How to cite this article**: Zhong, B.-L. *et al*. Elderly suicide trends in the context of transforming China, 1987–2014. *Sci. Rep*. **6**, 37724; doi: 10.1038/srep37724 (2016).

**Publisher's note:** Springer Nature remains neutral with regard to jurisdictional claims in published maps and institutional affiliations.

## Supplementary Material

Supplementary Information

## Figures and Tables

**Table 1:** Joinpoint analysis for elderly suicide mortality rates, China, 1987–2014.

Group	Period		Crude ESR			Standardized ESR		
	Beginning	End	Beginning	End	APC (95%CI)	Beginning	End	APC (95%CI)
National	1987	1998	76.6	82.2	0.5 (−2.4, 3.4)	76.7	85.9	0.8 (−2.1, 3.9)
	1998	2014	82.2	30.2	−6.0 (−7.6, −4.4)*	85.9	30.8	−6.2 (−7.8, −4.7)*
	1987	2014	76.6	30.2	−3.7 (−4.6, −2.7)*	76.7	30.8	−3.7 (−4.7, −2.7)*
Rural males	1987	1998	92.5	122.1	0.8 (−2.2, 3.9)	96.1	127.8	0.7 (−2.3, 3.7)
	1998	2014	122.1	40.3	−6.4 (−8.0, −4.7)*	127.8	40.5	−6.6 (−8.2, −5.0)*
	1987	2014	92.5	40.3	−3.8 (−4.8, −2.8)*	96.1	40.5	−4.0 (−5.0, −3.0)*
Rural females	1987	1998	79.1	87.4	1.6 (−1.4, 4.6)	83.5	89.2	1.5 (−1.4, 4.5)
	1998	2014	87.4	29.8	−6.9 (−8.6, −5.2)*	89.2	30.2	−7.0 (−8.7, −5.4)*
	1987	2014	79.1	29.8	−3.7 (−4.9, −2.6)*	83.5	30.2	−3.9 (−5.0, −2.7)*
Urban males	1987	2014	29.4	22.3	−0.7 (−3.6, 2.3)	32.3	21.9	−1.4 (−4.3, 1.6)
Urban females	1987	2014	45.5	15.3	−1.9 (−4.4, 0.7)	47.2	15	−2.1 (−4.7, 0.5)

ESR: elderly suicide rate, APC: annual percent change, *The APC is significantly different from 0 at level of significance 0.05.

**Table 2:** Joinpoint analysis for residence-gender-age elderly suicide mortality rates, China, 1987–2014.

Group	Period		Crude ESR		
	Beginning	End	Beginning	End	APC (95%CI)
Rural young-old men	1987	1998	87.3	104.1	0.8 (−1.8, 3.4)
	1998	2014	104.1	29.5	−8.3 (−9.8, −6.7)*
	1987	2014	87.3	29.5	−4.8 (−5.9, −3.6)*
Rural young-old women	1987	1998	70.5	77.5	1.2 (−1.7, 4.1)
	1998	2014	77.5	21.2	−8.8 (−10.6, −7.0)*
	1987	2014	70.5	21.2	−4.8 (−6.1, −3.5)*
Urban young-old men	1987	1999	25	16.5	−6.4 (−13.7, 1.5)
	1999	2002	16.5	54.2	50.5 (−62.9, 509.4)
	2002	2014	54.2	16.8	−10.7 (−15.5, −5.6)*
	1987	2014	25	16.8	−0.8 (−3.8, 2.3)
Urban young-old women	1987	1997	37.7	12.5	−8.4 (−13.9, −2.6)*
	1997	2005	12.5	49.4	14.1 (4.9, 24.1)*
	2005	2008	49.4	5.3	−39.7 (−75.9, 51.2)
	2008	2014	5.3	11.4	7.6 (−5.3, 22.4)
	1987	2014	37.7	11.4	−2.1 (−4.7, 0.5)
Rural old-old men	1987	1998	104.4	168.7	1.3 (−2.2, 5.1)
	1998	2014	168.7	55.9	−5.8 (−7.5, −4.0)*
	1987	2014	104.4	55.9	−3.4 (−4.4, −2.3)*
Rural old-old women	1987	1998	89.6	110.9	2.3 (−0.5, 5.2)
	1998	2014	110.9	40	−5.8 (−7.5, −4.0)*
	1987	2014	89.6	40	−3.3 (−4.4, −2.2)*
Urban old-old men	1987	2014	41.2	25.8	−1.6 (−4.6, 1.5)
Urban old-old women	1987	2014	63.6	18.4	−2.2 (−4.8, 0.5)
Rural oldest-old men	1987	2007	144.3	128	−3.2 (−4.6, −1.7)*
	2007	2010	128	256.8	28.4 (−12.3, 88.0)
	2010	2014	256.8	93.5	−22.5 (−30.4, −13.6)*
	1987	2014	144.3	93.5	−1.0 (−2.5, 0.6)
Rural oldest-old women	1987	2014	148.4	56.2	−1.9 (−3.5, −0.3)*
Urban oldest-old men	1987	2014	56.1	57.3	−1.3 (−4.2, 1.7)
Urban oldest-old women	1987	2014	50.3	30.6	−1.6 (−4.1, 0.9)

ESR: elderly suicide rate, APC: annual percent change, *The APC is significantly different from 0 at level of significance 0.05.
