# Supplementary material for: Elderly suicide trends in the context of transforming China, 1987–2014
Source: Sci Rep. 2016 Nov 25;6:37724. doi: 10.1038/srep37724 (PMC5123573; doi:10.1038/srep37724)
Supplement: Supplementary Information [file srep37724-s1.pdf]

Elderly suicide trends in the context of transforming China, 1987-2014

Bao-Liang Zhong[1,2], Helen F. K. Chiu[1*], and Yeates Conwell[3]

[1] Department of Psychiatry, The Chinese University of Hong Kong, Hong Kong SAR, China.

[2] Affiliated Mental Health Center, Tongji Medical College of Huazhong University of Science and Technology, Wuhan, Hubei, China.

[3] Department of Psychiatry, University of Rochester Medical Center, Rochester, New York, USA.

* Correspondence and requests for materials should be addressed to H.C. (email: helenchiu@cuhk.edu.hk)

Supplementary Table S1. Suicide rates (1/100000) by residence (rural/urban), gender, and 5-year old age-group in China, 1987-2014

Year	Gender	Residence	Old age-group				
			65-69	70-74	75-79	80-84	85+
1987	Male	1	21.84	29.76	35.58	54.10	56.08
1988	Male	1	24.63	32.55	49.57	70.03	69.19
1989	Male	1	27.35	34.65	46.78	73.96	104.90
1990	Male	1	20.45	32.63	50.28	62.12	103.91
1991	Male	1	22.34	34.10	47.02	64.35	72.24
1992	Male	1	19.80	30.58	34.01	48.48	71.01
1993	Male	1	16.53	20.33	34.28	49.27	51.79
1994	Male	1	13.66	21.94	32.96	43.82	53.33
1995	Male	1	13.58	24.88	37.78	48.97	48.37
1996	Male	1	14.36	23.32	33.73	39.59	64.98
1997	Male	1	13.56	23.65	32.22	45.91	39.29
1998	Male	1	13.54	21.46	31.41	41.65	43.01
1999	Male	1	13.53	20.95	33.69	48.11	53.14
2000	Male	1	13.59	19.54	26.48	36.51	38.25
2001	Male	1	11.51	13.76	22.61	40.33	37.50
2002	Male	1	45.59	65.84	93.22	127.78	193.48
2003	Male	1	35.91	51.41	72.97	87.53	89.34
2004	Male	1	21.24	36.29	33.10	62.02	53.64
2005	Male	1	44.20	78.90	111.95	139.39	105.99
2006	Male	1	11.21	18.16	21.89	34.75	57.37
2007	Male	1	8.26	17.02	16.72	27.75	28.64
2008	Male	1	7.52	9.02	10.85	16.00	16.82
2009	Male	1	11.54	15.31	23.72	31.76	34.34
2010	Male	1	17.27	29.25	39.88	68.88	117.48
2011	Male	1	9.86	15.97	24.14	42.83	45.08
2012	Male	1	11.83	17.35	25.83	32.92	35.19
2013	Male	1	13.79	17.15	23.39	34.90	40.02
2014	Male	1	15.06	19.37	24.08	28.56	57.34
1987	Female	1	30.93	46.97	55.38	79.01	50.25
1988	Female	1	20.24	32.60	42.90	57.57	74.58
1989	Female	1	22.72	32.15	37.56	53.39	73.18
1990	Female	1	17.54	23.76	39.36	56.62	51.52
1991	Female	1	18.35	28.22	39.54	50.59	53.67
1992	Female	1	19.35	26.58	32.81	48.65	62.37
1993	Female	1	15.34	20.36	28.84	46.36	44.00
1994	Female	1	12.06	21.43	28.89	32.50	46.14
1995	Female	1	15.98	22.06	29.61	39.33	41.63

1996	Female	1	13.52	21.49	27.79	35.14	30.90
1997	Female	1	9.75	16.73	26.55	34.39	39.60
1998	Female	1	13.91	25.24	24.39	37.31	30.65
1999	Female	1	14.65	20.49	23.61	34.04	29.58
2000	Female	1	12.24	16.37	24.57	27.50	33.84
2001	Female	1	11.22	13.99	20.76	26.00	27.60
2002	Female	1	25.33	46.65	67.10	78.80	98.90
2003	Female	1	26.46	32.34	53.13	73.18	66.83
2004	Female	1	22.61	27.92	32.99	36.03	59.65
2005	Female	1	38.76	62.93	83.91	119.78	96.72
2006	Female	1	8.08	10.87	15.72	22.42	28.84
2007	Female	1	8.43	11.59	13.28	19.91	19.53
2008	Female	1	4.78	5.89	8.63	13.16	12.16
2009	Female	1	8.41	11.34	17.97	28.06	29.05
2010	Female	1	13.88	20.98	26.65	48.47	71.95
2011	Female	1	9.85	12.19	24.07	32.57	34.94
2012	Female	1	9.19	12.56	18.79	25.18	30.50
2013	Female	1	9.58	12.41	15.12	25.10	22.28
2014	Female	1	10.65	12.56	15.42	23.29	30.61
1987	Male	2	74.32	106.47	97.51	120.35	144.28
1988	Male	2	65.75	107.72	111.51	148.18	173.44
1989	Male	2	70.06	108.54	124.02	170.65	159.99
1990	Male	2	64.05	104.38	116.61	165.48	196.56
1991	Male	2	76.06	136.72	127.40	154.56	166.50
1992	Male	2	75.81	131.46	137.96	155.44	162.23
1993	Male	2	75.41	125.65	136.61	172.75	150.94
1994	Male	2	76.83	139.25	127.27	172.80	147.87
1995	Male	2	69.33	112.01	110.70	163.11	131.18
1996	Male	2	64.00	94.00	124.07	148.04	139.19
1997	Male	2	77.91	127.41	126.99	155.53	143.03
1998	Male	2	79.76	137.95	154.08	202.01	173.22
1999	Male	2	66.83	109.06	131.36	149.98	153.03
2000	Male	2	68.49	106.27	111.17	154.25	147.80
2001	Male	2	70.43	101.18	122.03	161.01	133.65
2002	Male	2	54.48	77.66	107.23	132.65	137.11
2003	Male	2	59.24	88.55	109.67	130.79	101.42
2004	Male	2	33.47	53.12	53.33	123.32	94.48
2005	Male	2	39.82	46.00	74.23	96.54	77.12
2006	Male	2	29.67	45.01	58.04	87.89	83.46
2007	Male	2	34.27	51.69	67.46	103.51	127.97
2008	Male	2	27.32	31.46	47.19	68.40	106.10
2009	Male	2	26.48	43.21	64.07	90.31	130.25
2010	Male	2	29.19	51.44	88.71	151.03	256.81

2011	Male	2	29.57	41.69	50.99	104.13	148.44
2012	Male	2	21.64	29.90	47.50	64.93	92.21
2013	Male	2	25.47	37.05	53.66	73.17	95.03
2014	Male	2	25.31	35.46	49.16	67.51	93.54
1987	Female	2	61.03	83.15	76.10	114.77	148.39
1988	Female	2	57.98	78.16	81.48	102.17	129.19
1989	Female	2	58.41	76.75	87.41	96.09	44.33
1990	Female	2	43.30	61.45	61.74	82.49	102.49
1991	Female	2	64.52	89.43	96.96	121.24	131.87
1992	Female	2	54.50	74.03	80.98	84.69	119.30
1993	Female	2	61.45	95.78	96.76	94.05	93.95
1994	Female	2	57.90	97.68	91.24	108.66	113.94
1995	Female	2	54.73	85.57	91.41	125.13	126.02
1996	Female	2	57.23	91.25	88.07	119.38	126.11
1997	Female	2	64.48	89.00	97.75	125.40	111.77
1998	Female	2	63.51	95.94	103.06	124.29	83.31
1999	Female	2	58.55	86.23	89.56	115.16	117.37
2000	Female	2	48.92	80.65	93.77	115.46	113.50
2001	Female	2	54.85	82.85	105.72	125.34	156.57
2002	Female	2	31.67	47.62	77.86	72.73	101.58
2003	Female	2	50.33	64.13	77.86	72.73	101.58
2004	Female	2	26.86	37.10	64.86	66.86	114.32
2005	Female	2	38.35	39.33	56.30	71.93	111.71
2006	Female	2	22.91	27.82	40.08	51.59	69.61
2007	Female	2	22.67	30.23	48.85	75.13	81.08
2008	Female	2	13.60	22.76	36.40	41.28	42.47
2009	Female	2	20.80	27.34	43.80	74.78	91.90
2010	Female	2	24.81	37.31	52.30	79.49	159.70
2011	Female	2	21.47	28.87	42.39	60.52	63.76
2012	Female	2	17.83	23.20	39.79	43.79	58.72
2013	Female	2	18.59	24.79	35.27	48.38	58.92
2014	Female	2	17.52	26.50	35.53	46.62	56.19
